# Identifying prognostic lncRNAs based on a ceRNA regulatory network in laryngeal squamous cell carcinoma

**DOI:** 10.1186/s12885-021-08422-2

**Published:** 2021-06-15

**Authors:** Yong Shi, Dongli Yang, Yixiao Qin

**Affiliations:** 1 Department of Reproductive Medicine Center of the First Affiliated Hospital of Zhengzhou University, Zhengzhou University, Zhengzhou, 450000 China; 2 Department of Otorhinolaryngology Head and Neck Surgery, The First Hospital of Shanxi Medical University, Shanxi Medical University, Taiyuan, China

**Keywords:** LncRNA, CeRNA, Prognosis, Laryngeal squamous cell carcinoma

## Abstract

**Purpose:**

Growing evidence demonstrates that long non-coding RNAs (lncRNAs) play a crucial role as competing endogenous RNAs (ceRNAs) in tumor occurrence. The lncRNAs’ functions and clinical significance in laryngeal squamous cell carcinoma (LSCC) remain unclear. The study aims to reveal the lncRNA-associated ceRNA regulatory network of LSCC and clarify its clinical relevance.

**Methods:**

Here, we obtained LSCC transcriptome data from The Cancer Genome Atlas (TCGA) database and identified the differential expression profile of lncRNAs, miRNAs, and mRNAs by the EdgeR R package. The function enrichment analysis of mRNAs was performed using clusterProfiler R package and GSEA3.0. Then, we constructed a ceRNA network and prognosis model based on lncRNAs through bioinformatic methods. Moreover, we explored the functions of prognosis-related lncRNA in LSCC by CCK-8 and transwell assay.

**Results:**

1961 lncRNAs, 69 miRNAs, and 2224 mRNAs were identified as differentially expressed genes in LSCC tissues. According to the transcriptome differential expression profile, a ceRNA network containing 61 lncRNAs, 21 miRNAs, and 77 mRNAs was established. Then, four lncRNAs (AC011933.2, FAM30A, LINC02086, LINC02575) were identified from the ceRNA network to build a prognosis model for LSCC patients. And we found that LINC02086 and LINC02575 promoted the proliferation, migration, and invasion of LSCC cells while AC011933.2 and FAM30A inhibited these biological functions in vitro. Furthermore, we validated that LINc02086/miR-770-5p/SLC26A2 axis promoted migration in LSCC.

**Conclusion:**

Four lncRNAs of the ceRNA network were abnormally expressed and related to patient prognosis in LSCC. They played a significant role in the progress of LSCC via affecting the proliferation and metastasis of tumor cells.

**Supplementary Information:**

The online version contains supplementary material available at 10.1186/s12885-021-08422-2.

## Introduction

Laryngeal squamous cell carcinoma (LSCC) is a typical head and neck squamous cell carcinoma [1]. The conventional treatment for LSCC, including chemotherapy, radiation therapy, and surgical resection, have a satisfied curative effect on early-stage patients [2]. However, patients with advanced LSCC continuously have a low overall five-year survival rate [3]. Therefore, it is necessary to reveal the pathogenesis of LSCC and improve the level of diagnosis and treatment.

Non-coding RNAs (ncRNAs) are a type of RNA that cannot encode proteins, including small non-coding RNA (sncRNAs) and long non-coding RNA (lncRNAs) [4, 5]. MicroRNAs (miRNAs) that belong to sncRNAs and have been widely studied decreases the expression level of mRNAs by binding to its 3′-untranslated regions (3′-UTRs) to degrade mRNAs [6, 7]. Long non-coding RNAs (lncRNAs), the length of over 200 nucleotides (nt), can play significant regulation functions in several biological processes, including transcription, pretranscription, chromatin modification, translation, post-translation [8–10]. Currently, researchers found that the abnormal expression of lncRNAs is involved in the occurrence and development of malignant tumors [11]. For example, lncRNA HOTAIR is upregulated in many cancers and promotes the proliferation, migration, and invasion of tumor cells, including breast cancer, rectal cancer, pancreatic cancer, and kidney cancer [12–15]. Upregulated-LINC02410 may serve a diagnostic marker for rectal cancer [16].

The ceRNA hypothesis, a hot topic of lncRNA research, is that some lncRNAs sharing the same miRNA response elements (MREs) with mRNAs bind miRNAs competitively to block the interaction between miRNA and mRNA, thereby the expression level of mRNAs has been elevated [17, 18]. It has been reported that the ceRNA regulatory patterns extensively are present in many types of cancers [19]. For example, the lncRNA FAM225A upregulates ITGB3 by adsorbing miR-590-3p/miR-1275 to promote NPC cells’ proliferation and invasion [20]. lncRNA PVT1 acts as a ceRNA to adsorb miR-143 and upregulates the expression of HK2 to encourage the proliferation and metastasis of GBC cells [21]. However, the lncRNA-associated ceRNA network in LSCC remains unclear. So, this study aims to build a ceRNA regulatory network for a better understanding of lncRNA’s molecular mechanism in LSCC.

In this study, the LSCC transcriptome data was downloaded from the TCGA database, including the RNA expression profile of 111 tumor samples and 12 normal samples. Using bioinformatics tools, we constructed a ceRNA network containing 61 lncRNAs, 21 miRNAs, and 77 mRNAs. Meanwhile, a four-lncRNA prognosis model based on the ceRNA network was also established. Furthermore, we found that the four lncRNAs had apparent influences on the proliferation, migration, and invasion of LSCC cells in vitro. The above analysis and experiment results show that the four lncRNAs may serve prognosis biomarkers and become Therapeutic targets of LSCC in the future.

## Materials and methods

### Samples and patients from TCGA

Transcriptome sequencing data of patients with laryngeal squamous cell carcinoma (LSCC) were obtained from the TCGA database (https://portal.gdc.cancer.gov/), which contained 111 LSCC samples and 12 normal samples. Besides, the corresponding clinical characteristics of LSCC patients’ overall survival (OS) were obtained from the TCGA database.

### Identification of differentially expressed lncRNA, mRNA, and miRNA

Ensemble IDs of the genes were transformed into gene symbols based on GENCODE (https://www.gencodegenes.org/human/). Differentially expressed lncRNAs (DElncRNAs), differentially expressed mRNAs (DEmRNAs), and differentially expressed miRNA (DEmiRNAs) between LSCC samples and normal samples were identified by the EdgeR R package [22]. We regarded lncRNAs, mRNAs, and miRNAs as DElncRNAs, DEmRNAs, and DEmiRNAs when they met these criteria (*p* < 0.05, and |log2 FC| > 2).

### Functional enrichment analysis

Through clusterProfiler R package [23], We performed Gene Ontology (GO) and Kyoto Encyclopedia of Genes and Genomes (KEGG) analysis [24]. GSEA analysis was performed by GSEA3.0 (http://software.broadinstitute.org/gsea/index.jsp), and the cut-off criteria for gene sets were set to adjust *p* < 0.05.

### Construction of lncRNA-miRNA-mRNA ceRNA network

DElncRNAs, DEmiRNAs, and DEmRNAs were used to establish the ceRNA network. Firstly, we screened the DElncRNA-DEmiRNA pairs using Starbase [25]. Secondly, TargetScan [26], miRDB [27], and miRwalk [28] were used to screen the DEmiRNA-DEmRNA pairs. When DEmiRNA-DEmRNA pairs were predicted in all three databases, we considered candidates for constructing the ceRNA network. Ultimately, Cytoscape 3.6.1 was used to build a lncRNA-related ceRNA network [29].

### Screening of LSCC prognostic signatures

Survival analysis and univariate cox regression analysis were performed to explore the correlation between the lncRNAs from the ceRNA network and OS of LSCC patients by the survival R package [30]. Then prognosis model was built by multivariate regression analysis. And the following formula was used to calculate the prognostic risk score of LSCC patients: Risk score = βlncRNA1 * explncRNA1 + βlncRNA2 * explncRNA2 + … + βlncRNAn * explncRNAn (‘β’ is the regression coefficient of lncRNAs and ‘exp’ is the expression of corresponding lncRNAs). Through the survivalROC R package, a receiver operating characteristic (ROC) curve was drawn to assess the prognosis model’s accuracy.

### Cell culture and transfection

Human laryngeal cancer cell lines TU177, Hep-2 were purchased from Bena Culture Collection (Beijing, China). All cells were cultured in Dulbecco’s Modified Eagle Medium (DMEM; Gibco, USA) with 10% Fetal Bovine Serum (FBS; Gibco, USA) at 37 °C and 5%CO_2_. The cells were transfected with prognosis related-lncRNA using Lipofectamine™ 3000 (Thermo Fisher Scientific, USA).

### Quantitative real-time PCR

According to the manufacturer’s protocol, the total RNA was extracted from cells by TRIzol reagent (Invitrogen, CA, USA). qRT-PCR analysis on lncRNA was performed using HiScript® II Q Select RT SuperMix for qPCR Kit (vazyme, Nanjing, China) and ChamQ Universal SYBR qPCR Master Mix (vazyme, Nanjing, China). 18S was used as the endogenous control.

### CCK-8 assay

Cell proliferation was examined by Cell Counting Kit-8 (CCK-8; GLPBIO, USA). The cells were incubated in the 96-well plates (1 × 10^3^ cells per well) for 24 h, and then 10 μl CCK-8 reagents were added to each well. Cell viability was determined by detecting the absorbance at 450 nm.

### Cell migration and invasion assay

Transwell chambers with 8.0 μM pore polycarbonate membrane insert (Corning, USA) assessed cell migration and invasion abilities. For cell invasion assay, 40 μL matrigel solution (Matrigel: medium = 1: 4) was added to transwell inserts and solidified for 3 h at 37 °C. Then 500 μL DMEM with 10% FBS was added to the lower chamber. Cells were resuspended with serum-free medium and plated into transwell inserts at 1 × 10^5^ cells/well. The cells on the filter’s upper surface were removed after they were cultured at 37 °C for 24–72 h. For cell migration assay, the matrigel solution was not needed on the inserts, and other steps were as same as invasion assay. Cells on the lower surface of the filter were fixed with 4% paraformaldehyde for 20 mins and then washed twice with PBS before they were stained with 1% crystal violet solution for 10 mins. The stained cells were counted by a microscope.

### Statistical analysis

The statistical analysis software included R 3.4.3 and SPASS 22.0. All measurement data were presented as mean ± SD. Two-tailed Student’s t-test was used to compare two groups. *P*-value < 0.05 was considered statistically significant.

## Results

### Identification of DElncRNAs, DEmiRNAs, and DEmRNAs

**Fig. 1:**
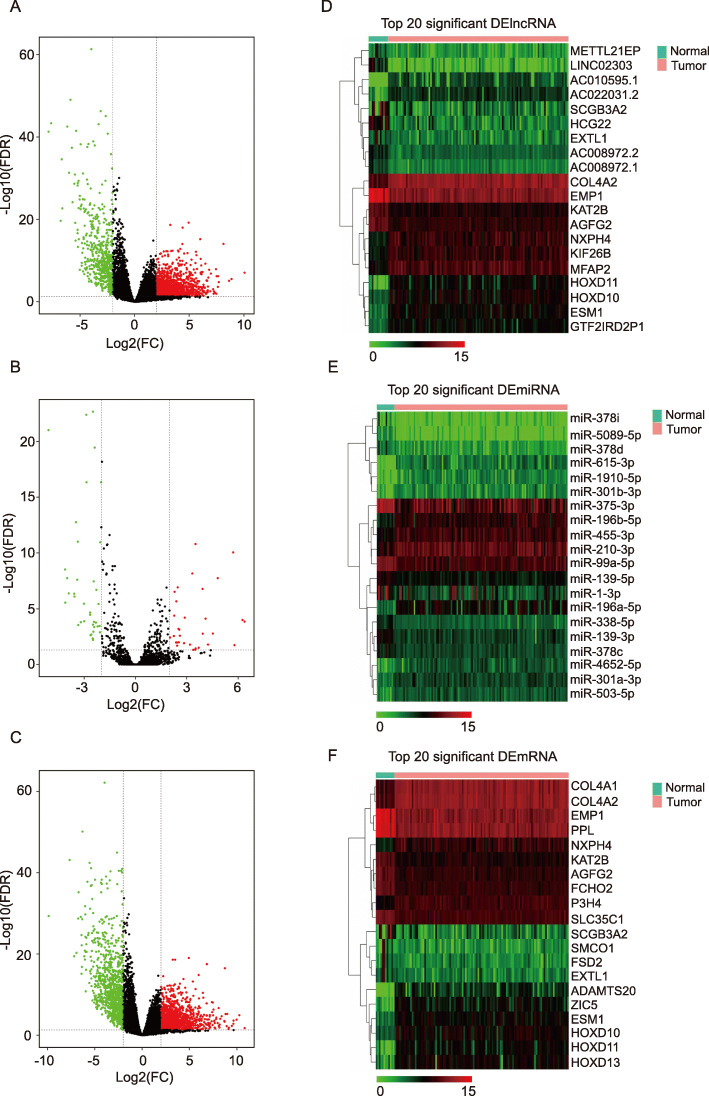
Volcano plots and heatmaps show DElncRNAs, DEmiRNAs, and DEmRNAs. (**A-C)** Volcano plots for DElncRNAs, DEmiRNAs, and DEmRNAs. (**E-F)** heatmaps for DElncRNAs, DEmiRNAs, and DEmRNAs

Considering the cut points of |log2-fold change| > 2 and *p* < 0.05, 1961 DElncRNAs, 69 DEmiRNAs, and 2224 DEmRNAs were identified to be differentially expressed genes in LSCC tissues. Among them, 1398 lncRNAs, 32 miRNAs, and 1285 mRNAs were high expression genes, while 563 lncRNAs, 37 miRNAs, and 939 mRNAs were the low expression genes in LSCC (Supplementary Material 1). The differences in the expression of lncRNAs, miRNAs, and mRNAs in LSCC were demonstrated on volcano plots (Fig. 1A, B, C). The top 20 DElncRNAs, DEmiRNAs, DEmRNAs expression profiles in heatmaps, respectively (Fig. 1D, E, F).

### DEmRNAs functional enrichment analysis

**Fig. 2:**
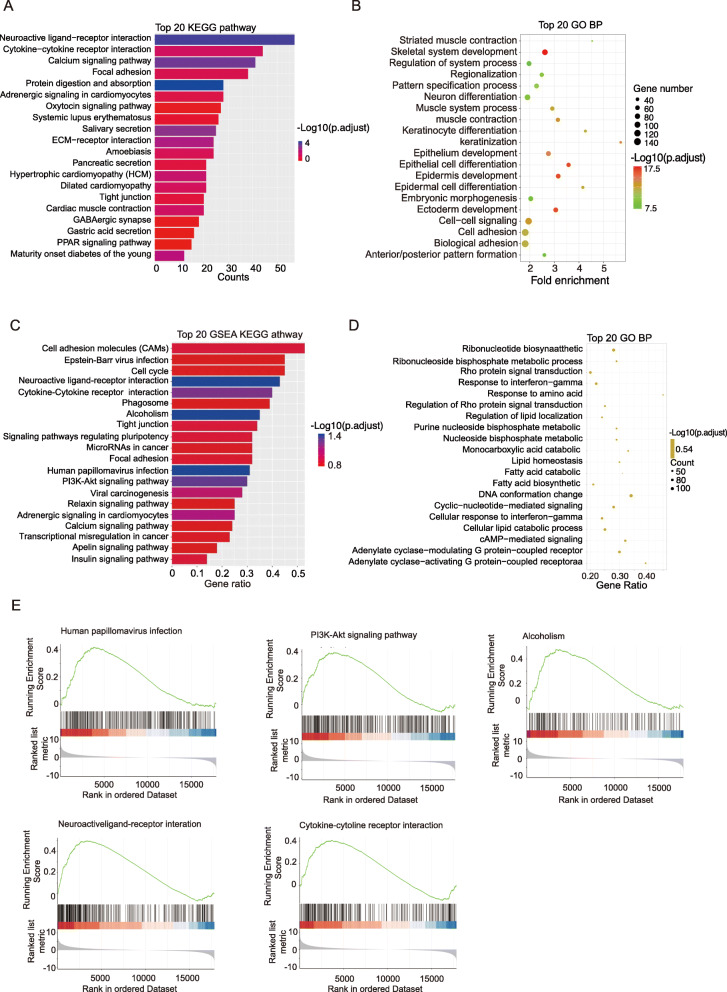
GO and KEGG pathway function enrichment analysis for DEmRNAs. **A** Top 20 KEGG pathways based on the *p*-value. **B** Top 20 GO BP terms based on *p* values. **C** Top 20 KEGG-GSEA pathway based on adjust *p*-values. **D** Top 20 GO BP-GSEA terms based on adjust *p*-values. **E** Five statistically significant KEGG-GSEA pathways with adjust *p* < 0.05

For exploring the function and molecular mechanism of DEmRNAs in the occurrence and development of laryngeal squamous cell carcinoma, we performed GO and KEGG enrichment analysis on DEmRNAs (Supplementary Material 2). The top 20 KEGG pathways and top 20 GO biological process terms (*p* < 0.05), sorted by the *p*-values, were chosen for Bar graph and bubble graph (Fig. 2A, B). Among these pathways and biological process terms, some terms containing ECM-receptor interactions, Tight junction, and PPAR signaling pathway are correlated to the proliferation and metastasis of tumors. In addition, KEGG-GSEA analysis showed that DEmRNAs gene sets enrich in Human papillomavirus infection, PI3K-Akt signaling pathway, Alcoholism, Neuroactive ligand-receptor interaction, Cytokine-cytokine receptor interaction (Fig. 2C, E; adjust *p* < 0.05; Supplementary Material 3). GO biological process GSEA analysis results had no statistical significance (Fig. 2D; adjust *p* > 0.05).

### Construction of the lncRNA-miRNA-mRNA ceRNA network

**Fig. 3:**
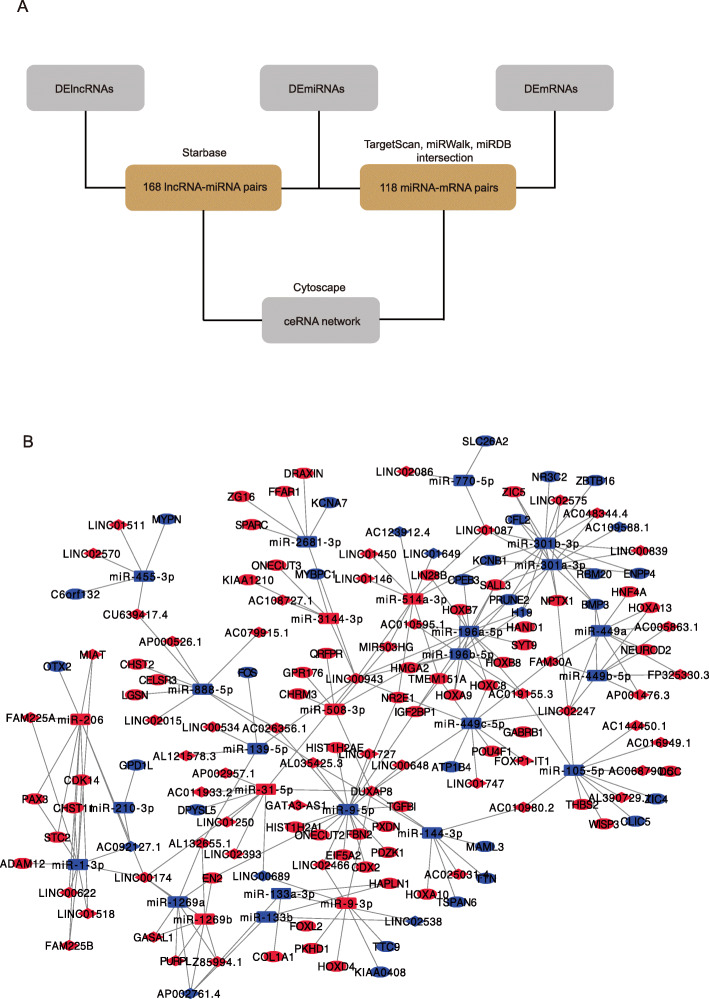
Construction of ceRNA network in LSCC. **A** The workflow of building ceRNA network. **B** CeRNA network regulation model. The diamond represents lncRNA. Rectangle represents miRNA. Oval represents mRNA. Red represents high expression in cancer tissues. Blue represents low expression in cancer tissues

To explore the ceRNA Molecular regulatory network centered on lncRNA in LSCC, we constructed the ceRNA network according to the workflow (Fig. 3A). First, the interactions between lncRNAs and miRNAs were predicted by the starbase database among the DElncRNAs and DEmiRNAs. We obtained 168 lncRNA-miRNA pairs (Supplementary Material 4). Next, the interactions between miRNAs and mRNA were screened by TargetScan, miRDB, and miRWalk among the DEmiRNAs and DEmRNAs. We obtained 118 miRNA-mRNA pairs that all interactions exist in three databases (Supplementary Material 4). Then we used Cytoscape software to construct a lncRNA-miRNA-mRNA ceRNA network consisting of 61 lncRNAs, 21 miRNAs, and 77 mRNAs with a total of 179 interactions, based on the lncRNA-miRNA pairs and the miRNA-mRNA pairs (Fig. 3B).

### Screening lncRNA-associated prognosis factors based on ceRNA network

**Fig. 4:**
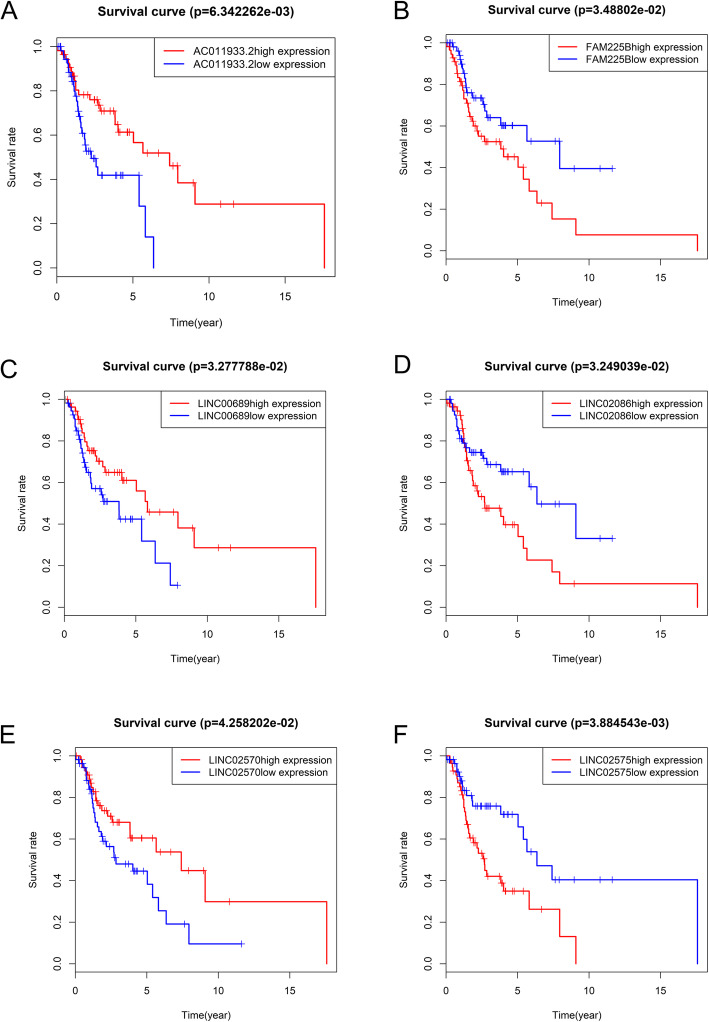
Kaplan-Meier survival curves of six lncRNAs (log-rank *p* < 0.05). One hundred eleven LSCC patients were divided into two groups (high expression and low expression) according to the median of relative expression value

**Fig. 5:**
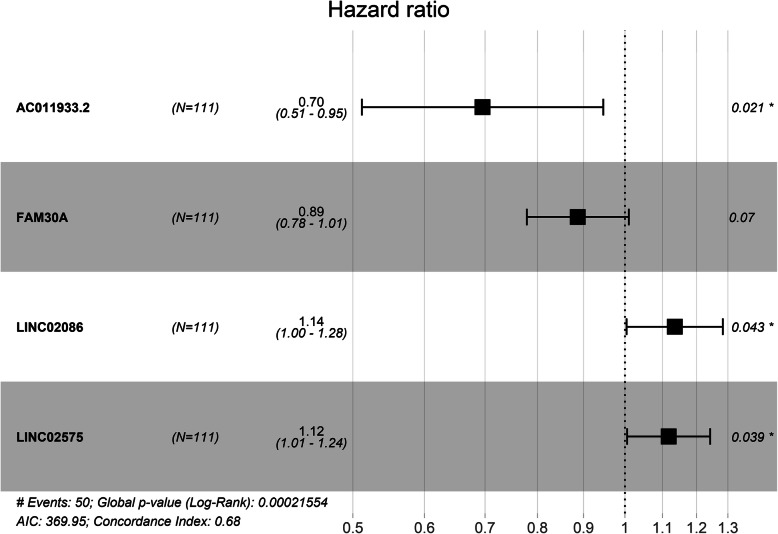
Forecast plots for the four prognosis-related lncRNAs

**Fig. 6:**
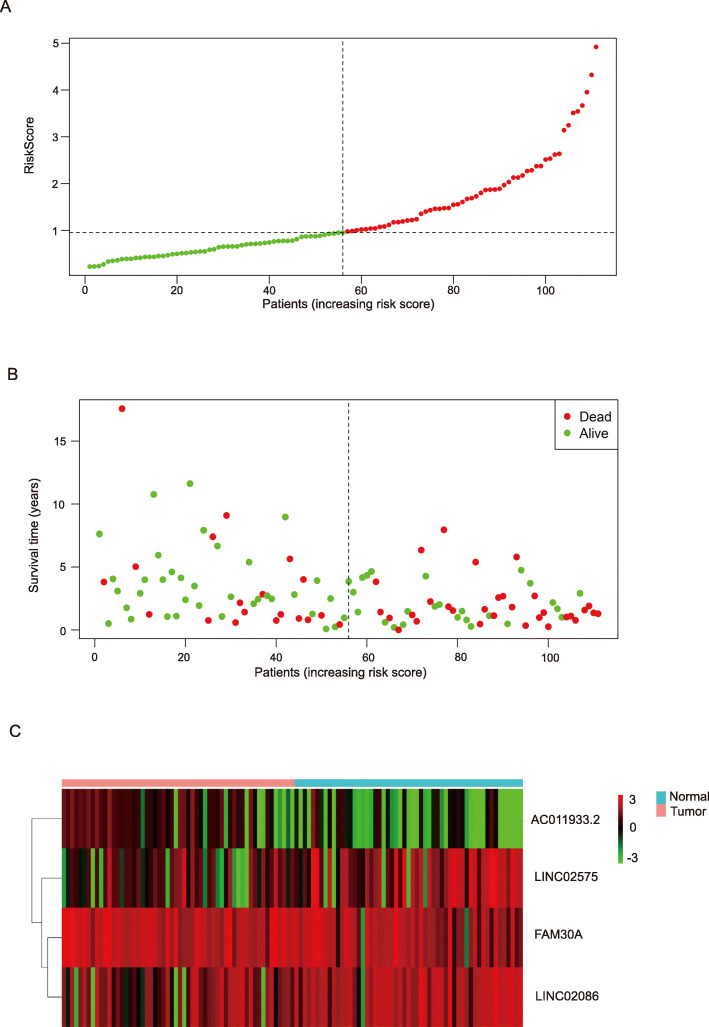
Risk score of all patients with LSCC. **A** Risk score plots of 111 patients. **B** survival state of 111 patients. **C** Risk score heatmap for 111 patients

**Fig. 7:**
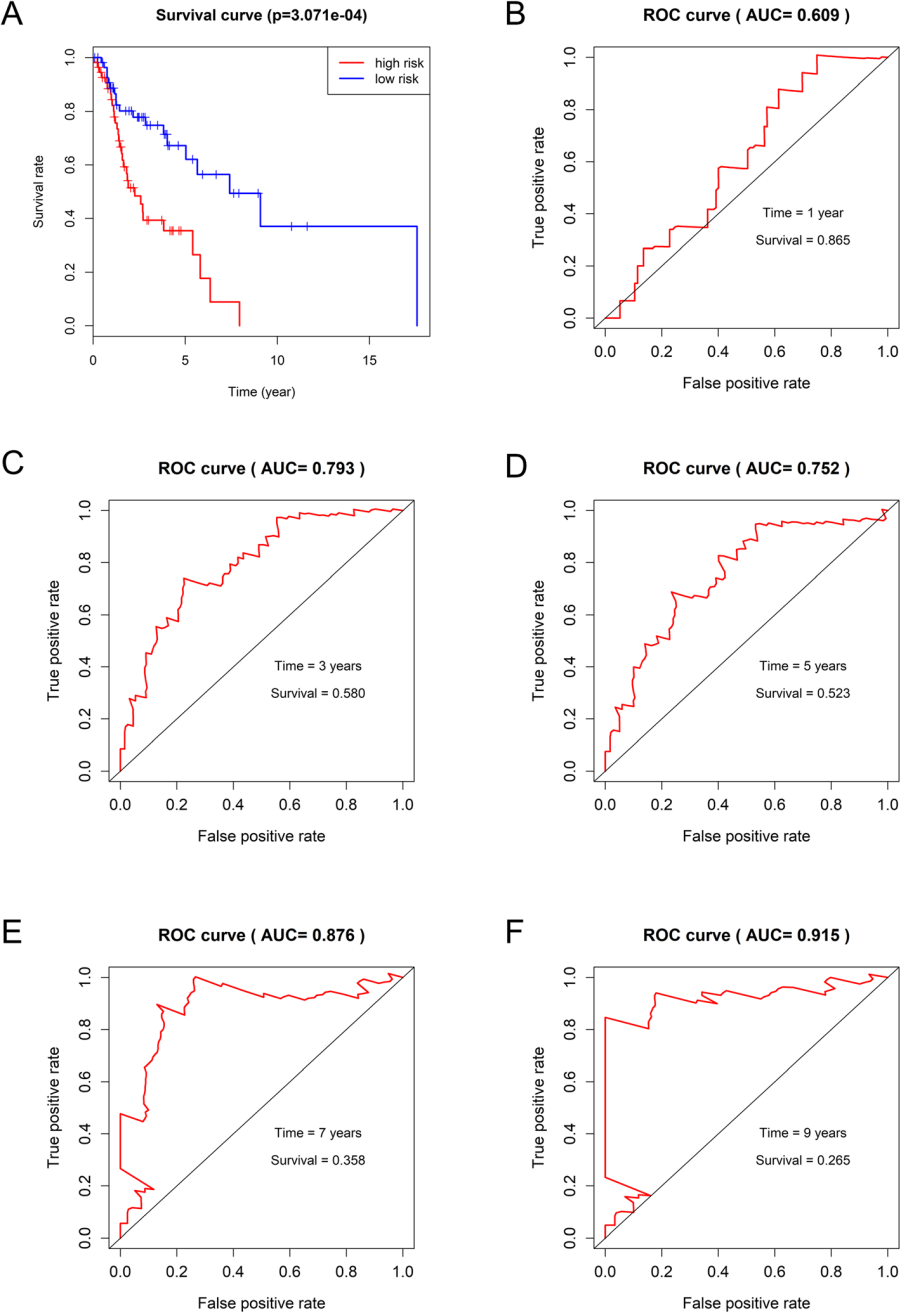
Examining the specificity and sensitivity of the prognosis model. **(A)** Kaplan-Meier survival curves. One hundred eleven LSCC patients were divided into two groups (high risk and low risk) according to the median of the risk score. **(B-F)** ROC curves

Kaplan-Meier analysis results showed that six lncRNAs, including AC011933.2, LINC00689, LINC02570, FAM225B, LINC02086, and LINC02575 were closely associated with overall survival (OS) of patients (Fig. 4; *p* < 0.05). furthermore, Univariate cox regression analysis and multivariate cox regression analysis based on DElncRNAs from ceRNA network were performed to construct a prognosis model as follows: risk score = relative expression of AC011933.2*(− 0.36287) + relative expression of FAM30A*(− 0.12015) + relative expression of LINC02086*(0.12687) + relative expression of LINC02575*(0.11148). (Fig. 5) was a forest plot for the four lncRNAs consisted of the prognosis model. The survival risk score of all LSCC patients was presented in (Fig. 6A, C). (Fig. 6B) was a survival state graph for all LSCC patients. For detecting the specificity and sensitivity of the model. The Kaplan-Meier analysis result showed that the high-risk group’s survival rate tended to lower than the low-risk group (Fig. 7A; *p* < 0.05). The ROC analysis results showed that the area under curve (AUC) value was 0.609 in the 1st year, 0.793 in the 3rd year, 0.752 in the 5th year, 0.876 in the 7th year, 0.915 in the 9th year, which indicated that the cox risk model has high specificity and sensitivity (Fig. 7B-F).

### Prognosis-related lncRNAs affected the proliferation, migration, and invasion of LSCC cells

**Fig. 8:**
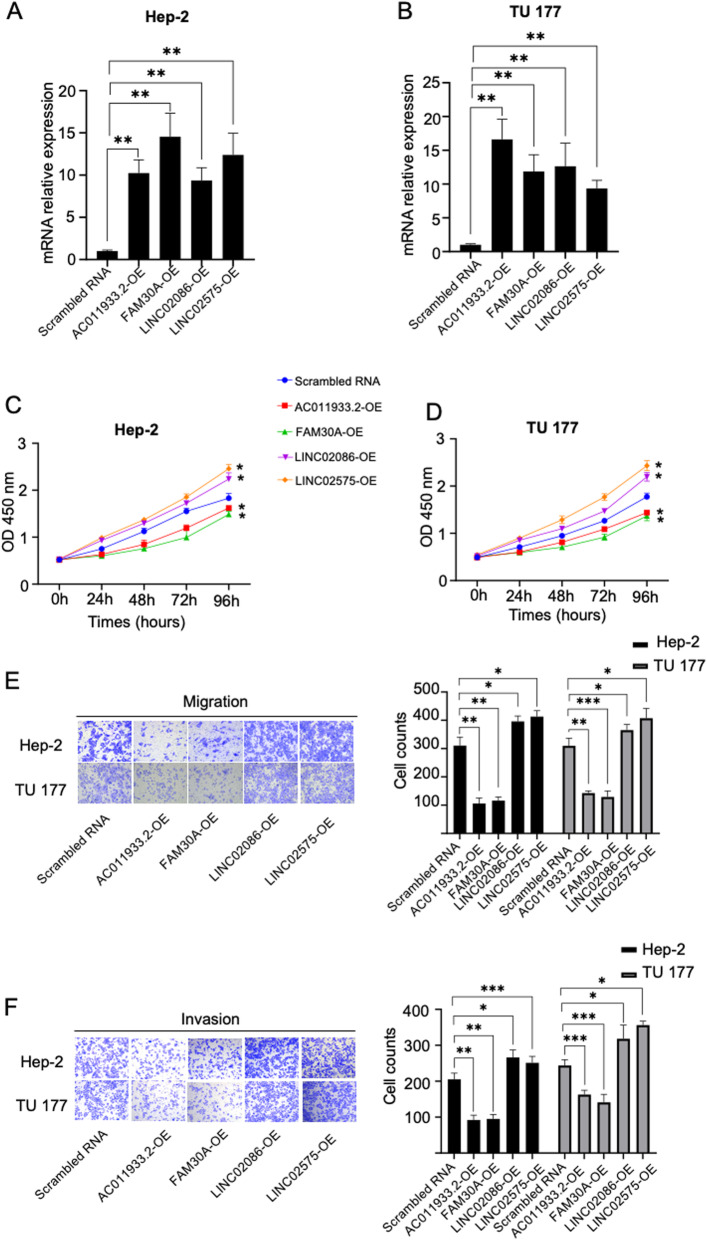
The effection of prognosis-related lncRNAs on LSCC cells. **(A, B)** We examined mRNA level by qPCR after Hep-2 and TU 177 were transfected plasmid containing prognosis-related lncRNA fragment. AC011933.2-OE, FAM30-OE, LINC02086-OE, and LINC02575-OE represent overexpression groups of AC011933.2, FAM30, LINC02086, and LINC02575, respectively. **(C, D)** We detected cell viability at five-time points (0 h, 24 h, 48 h, 72 h, 96 h) respectively after Hep-2 and TU 177 were transfected. **(E, F)** LSCC cells (Hep-2, TU 177) migration and invasion abilities were determined 48 h after transfection. * indicating *p* < 0.05. ** indicating *p* < 0.01. *** indicating *p* < 0.001

To understand the role of prognosis-related lncRNAs in the oncogenesis of LSCC, we elevated the expression level of prognosis-related lncRNAs by transfected pcDNA3.1(+)-prognosis-related lncRNAs into LSCC cells. The results showed that all four lncRNAs expression increased in the experimental groups compared to the control groups (Fig. 8A, B). The CCK-8 assay demonstrated that both LINC02086 and LINC02575 promoted Hep-2 and TU 177 proliferation while both AC011933.2 and FAM30A inhibited Hep-2 and TU 177 proliferation (Fig. 8C, D; *p* < 0.05). Then, the migration and invasion were detected by transwell assay. The results revealed that both LINC02086 and LINC02575 promoted Hep-2 and TU 177 cell migration and invasion while both AC011933.2 and FAM30A inhibited Hep-2 and TU 177 migration and invasion (Fig. 8E, F; *p* < 0.05).

### LINC02086 promotes migration by miR-770-5p/SLC26A2 axis in LSCC

**Fig. 9:**
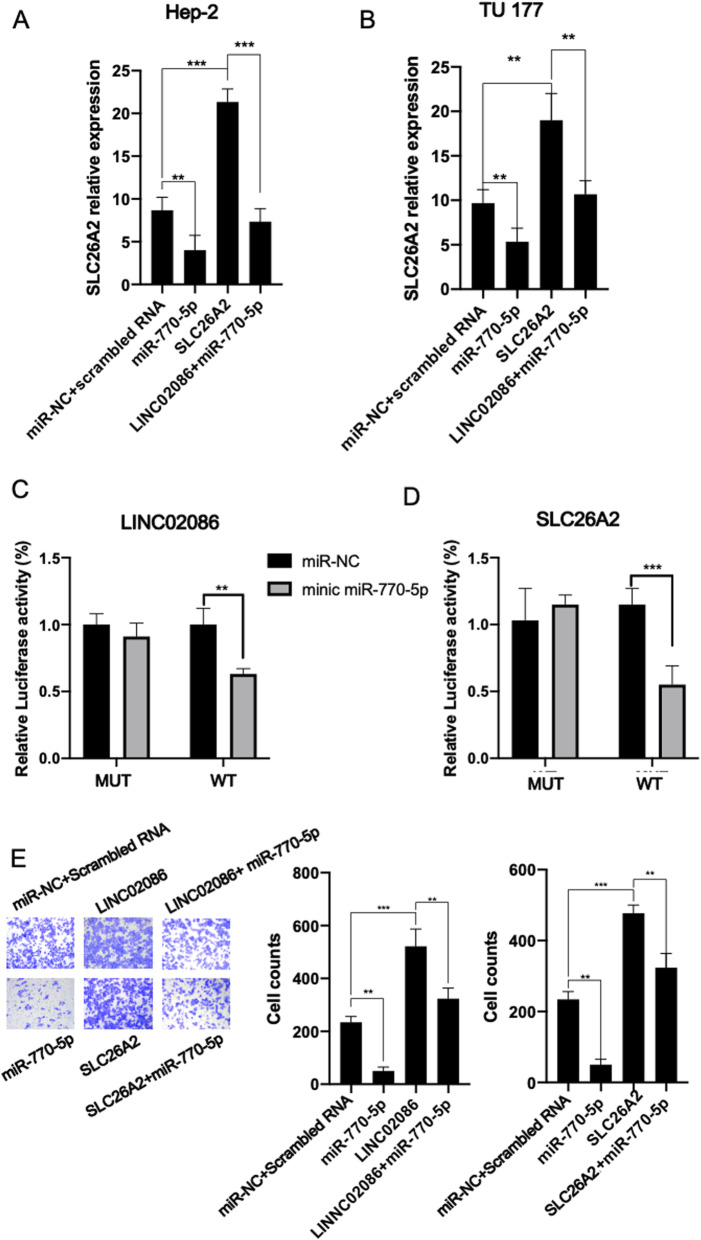
The affection of LINC02086/miR-770-5p/SLC26A2 axis on LSCC cells. **(A, B)** We examined SLC26A2 mRNA level by qPCR after Hep-2 and TU 177 were transfected plasmid containing SLC26A2 or LINC02086 fragment and minic miR-770-5p. **(C, D)** Dual-luciferase report analysis (Hep-2) for LINC02086-miR-770-5p and SLC26A2-miR-770-5p. **(E)** Hep-2 migration ability was examined 48 h after transfection. * indicating *p* < 0.05. ** indicating *p* < 0.01. *** indicating *p* < 0.001

In order to validate the ceRNA model, LINC02086/miR-770-5p/SLC26A2 axis was extracted from the ceRNA network for experimental verification. Firstly, SLC26A2 mRNA level was significantly increased after the Hep-2 and TU 177 cells were transfected with LINC02086 overexpression vector compared with the control group, while SLC26A2 mRNA level was decreased after the Hep-2 and TU 177 cells were transfected with LINC02086 and minic miR-770-5p (Fig. 9A-B). Dual-luciferase report analysis showed that the activity of Dual-luciferase was significantly decreased in the wild-type LINC02086 and SLC26A2 groups after transfection with minic miR-770-5p (Fig. 9C-D). The migration experiments showed that miR-770-5p could partially reverse the effect of LINC02086 on the migration of larynx carcinoma cells, while SLC26A2 could weaken the effect of miR-770-5p on the migration of larynx carcinoma cells (Fig. 9E). These results suggested that LINC02086 promoted the up-regulation of SLC26A2 by adsorbing miR-770-5p, thereby promoting the migration of larynx carcinoma cells.

## Discussion

LSCC, a type of common head and neck squamous cell carcinomas, causes a severe threat to peoples’ health all over the world [31]. As the incidence of laryngeal squamous cell carcinoma is increasing year by year, it is urgent to clarify the pathogenesis and identify significant prognosis biomarkers for improving the current treatment to LSCC. Our study demonstrated that a four-lncRNA prognosis model based on the ceRNA network had a sufficient ability to predict the prognosis of LSCC. Moreover, the four lncRNAs played a significant role in the proliferation and metastasis of LSCC. Therefore, these prognosis-related lncRNAs may serve as a new therapeutic target in the future.

LncRNA plays a vital role in the occurrence and development of tumors. Recently, more and more studies have shown that the ceRNA molecular regulation model is a common way for lncRNA to promote or inhibit tumor growth [32]. For example, ABHD11-AS1 serves as a competitive endogenous RNA to upregulate STAT3 by sponging miR-1301-3p in PTC [33]. Linc-DYNC2H1–4 promotes EMT and CSC phenotypes by acting as a sponge of miR-145 in pancreatic cancer cells [34]. However, most previous studies are limited to a single molecular regulation axis, and there is a lack of comprehensive and in-depth research on molecular regulation networks. As we know, the interaction between lncRNA and miRNA is not the only correspondence relationship. One lncRNA may have many different MREs, and other lncRNAs may also have the same MREs. For example, lnc HOTAIR can act as a molecular sponge for both miR-206 and miR-124 [15, 35]. Both lncRNA TUG1 and lncRNA PVT1 can interact with miR-145 [36, 37]. Similarly, the interaction between miRNA and mRNA is not the only correspondence relationship. Therefore, it is helpful for a comprehensive understanding of the molecular mechanism of lncRNA to elucidate the regulatory network of lncRNA-miRNA-mRNA.

Our study determined 1961 lncRNAs, 69 miRNAs, and 2224 mRNAs with abnormal expression genes in LSCC according to the transcriptome sequencing data derived from the TCGA. Then, functional enrichment analysis, including GO, KEGG, and GSEA, were performed by bioinformatics tools. We found that DEmRNAs were enriched in some tumor-related biological processes or pathways such as ECM-receptor interactions, Tight junction, PPAR signaling pathway, Human papillomavirus infection, PI3K-Akt signaling pathway. Next, a lncRNA-miRNA-mRNA ceRNA network was constructed according to DElncRNAs, DEmRNAs, and DEmiRNAs, and it included 61 lncRNAs, 21 miRNAs, and 77 mRNAs. Furthermore, we extracted a four-lncRNA (AC011933.2, FAM30A, LINC02086, LINC02575) prognosis model from the ceRNA network. These results indicate that the ceRNA network is useful for uncovering the molecular mechanism of lncRNA in LSCC and may become prognosis biomarkers of LSCC.

Several risk score systems were established to predict LSCC patients’ prognosis in previous studies [38, 39]. However, all these prognosis biomarkers based on the predicting model have not been verified by experiments in vitro. Our study constructed a four-lncRNA prognosis model and confirmed the biological functions of the four lncRNAs in LSCC.

The four lncRNAs’ functions in the progress of LSCC remain unclear because of the few associated studies. In our research, we found that the expression of AC011933.2 and FAM30A is abnormal in LSCC, and they have a negative correlation with the OS of LSCC patients. On the contrary, LINC02086 and LINC02575 are positively correlated with the overall survival of LSCC patients. Furthermore, the four lncRNAs were found to promote or inhibit LSCC cell proliferation, migration, and invasion in vitro. Therefore, the four lncRNA may be considered as new tumor suppressor genes and oncogenes that deserve further research.

Inevitably, our research has several innate limitations that need to be addressed. Although the bioinformatics process of constructing the ceRNA network was designed reasonably, and experiments were performed in vitro to verify the functions of prognosis-associated lncRNAs, our study’s main disadvantage was the lack of validation of the lncRNA-miRNA-mRNA molecular regulatory axis extracted from the ceRNA network. Besides, our cox risk model was based on data derived from the TCGA database, and clinical validation of larger samples is necessary. Despite the above drawbacks, our study results show that our cox risk model with 4 lncRNAs play a significant role in the occurrence and development of LSCC.

## Conclusion

In conclusion, we have identified a lncRNA-related prognosis model that can effectively predict OS in LSCC. Moreover, we verified these lncRNAs could influence the biological function of LSCC cells.

## Supplementary Information

**Additional file 1: Supplementary** **Material 1.** The differentially expressed genes list of lncRNAs, mRNAs, and miRNAs in LSCC.

**Additional file 2: Supplementary Material 2.** All GO and KEGG items of DEmRNAs.

**Additional file 3: Supplementary Material 3.** All GO_GSEA and KEGG_GSEA items of DEmRNAs.

**Additional file 4: Supplementary Material 4.** The list of miRNA-mRNA pairs and lncRNA-miRNA pairs.

## Data Availability

All data generated or analysed during this study are obtained from TCGA database (https://portal.gdc.cancer.gov/).

## References

[1] Siegel RL, Miller KD, Jemal A (2019). Cancer statistics, 2019. CA Cancer J Clin.

[2] Baird BJ, Sung CK, Beadle BM, Divi V (2018). Treatment of early-stage laryngeal cancer: a comparison of treatment options. Oral Oncol.

[3] Miller KD, Nogueira L, Mariotto AB, Rowland JH, Yabroff KR, Alfano CM, Jemal A, Kramer JL, Siegel RL (2019). Cancer treatment and survivorship statistics, 2019. CA Cancer J Clin.

[4] Romano G, Veneziano D, Acunzo M, Croce CM (2017). Small non-coding RNA and cancer. Carcinogenesis.

[5] Slack FJ, Chinnaiyan AM (2019). The role of non-coding RNAs in oncology. Cell.

[6] Lu TX, Rothenberg ME (2018). MicroRNA. J Allergy Clin Immunol.

[7] Dong H, Lei J, Ding L, Wen Y, Ju H, Zhang X (2013). MicroRNA: function, detection, and bioanalysis. Chem Rev.

[8] Ulitsky I, Bartel DP (2013). lincRNAs: genomics, evolution, and mechanisms. Cell.

[9] Kopp F, Mendell JT (2018). Functional classification and experimental dissection of long noncoding RNAs. Cell.

[10] St Laurent G, Wahlestedt C, Kapranov P (2015). The landscape of long noncoding RNA classification. Trends Genet.

[11] Bhan A, Soleimani M, Mandal SS (2017). Long noncoding RNA and Cancer: a new paradigm. Cancer Res.

[12] Gupta RA, Shah N, Wang KC, Kim J, Horlings HM, Wong DJ, Tsai M-C, Hung T, Argani P, Rinn JL (2010). Long non-coding RNA HOTAIR reprograms chromatin state to promote cancer metastasis. Nature.

[13] Kim K, Jutooru I, Chadalapaka G, Johnson G, Frank J, Burghardt R, Kim S, Safe S (2013). HOTAIR is a negative prognostic factor and exhibits pro-oncogenic activity in pancreatic cancer. Oncogene.

[14] Kogo R, Shimamura T, Mimori K, Kawahara K, Imoto S, Sudo T, Tanaka F, Shibata K, Suzuki A, Komune S (2011). Long noncoding RNA HOTAIR regulates polycomb-dependent chromatin modification and is associated with poor prognosis in colorectal cancers. Cancer Res.

[15] Pan Y, Wu Y, Hu J, Shan Y, Ma J, Ma H, Qi X, Jia L (2018). Long noncoding RNA HOTAIR promotes renal cell carcinoma malignancy through alpha-2, 8-sialyltransferase 4 by sponging microRNA-124. Cell Prolif.

[16] Zhao Y, Du T, Du L, Li P, Li J, Duan W, Wang Y, Wang C (2019). Long noncoding RNA LINC02418 regulates MELK expression by acting as a ceRNA and may serve as a diagnostic marker for colorectal cancer. Cell Death Dis.

[17] Tay Y, Rinn J, Pandolfi PP (2014). The multilayered complexity of ceRNA crosstalk and competition. Nature.

[18] Sanchez-Mejias A, Tay Y (2015). Competing endogenous RNA networks: tying the essential knots for cancer biology and therapeutics. J Hematol Oncol.

[19] Qi X, Zhang D-H, Wu N, Xiao J-H, Wang X, Ma W (2015). ceRNA in cancer: possible functions and clinical implications. J Med Genet.

[20] Zheng Z-Q, Li Z-X, Zhou G-Q, Lin L, Zhang L-L, Lv J-W, Huang X-D, Liu R-Q, Chen F, He X-J (2019). Long noncoding RNA FAM225A promotes nasopharyngeal carcinoma tumorigenesis and metastasis by acting as ceRNA to sponge miR-590-3p/miR-1275 and upregulate ITGB3. Cancer Res.

[21] Chen J, Yu Y, Li H, Hu Q, Chen X, He Y, Xue C, Ren F, Ren Z, Li J (2019). Long non-coding RNA PVT1 promotes tumor progression by regulating the miR-143/HK2 axis in gallbladder cancer. Mol Cancer.

[22] Robinson MD, McCarthy DJ, Smyth GK (2010). edgeR: a Bioconductor package for differential expression analysis of digital gene expression data. Bioinformatics.

[23] Yu G, Wang LG, Han Y, He QY (2012). clusterProfiler: an R package for comparing biological themes among gene clusters. OMICS.

[24] Kanehisa M (1997). A database for post-genome analysis. Trends Genet.

[25] Li J-H, Liu S, Zhou H, Qu L-H, Yang J-H (2014). starBase v2.0: decoding miRNA-ceRNA, miRNA-ncRNA and protein-RNA interaction networks from large-scale CLIP-Seq data. Nucleic Acids Res.

[26] Agarwal V, Bell GW, Nam J-W, Bartel DP. Predicting effective microRNA target sites in mammalian mRNAs. eLife. 2015;4. 10.7554/eLife.05005. 10.7554/eLife.05005 PMC4532895 26267216

[27] Chen Y, Wang X (2020). miRDB: an online database for prediction of functional microRNA targets. Nucleic Acids Res.

[28] Sticht C, De La Torre C, Parveen A, Gretz N (2018). miRWalk: an online resource for prediction of microRNA binding sites. PLoS One.

[29] Otasek D, Morris JH, Boucas J, Pico AR, Demchak B (2019). Cytoscape automation: empowering workflow-based network analysis. Genome Biol.

[30] Rizvi AA, Karaesmen E, Morgan M, Preus L, Wang J, Sovic M, Hahn T, Sucheston-Campbell LE (2019). gwasurvivr: an R package for genome-wide survival analysis. Bioinformatics.

[31] Cossu AM, Mosca L, Zappavigna S, Misso G, Bocchetti M, De Micco F, Quagliuolo L, Porcelli M, Caraglia M, Boccellino M (2019). Long Non-coding RNAs as Important Biomarkers in Laryngeal Cancer and Other Head and Neck Tumours. Int J Mol Sci.

[32] Schmitz SU, Grote P, Herrmann BG (2016). Mechanisms of long noncoding RNA function in development and disease. Cell Mol Life Sci.

[33] Wen J, Wang H, Dong T, Gan P, Fang H, Wu S, Li J, Zhang Y, Du R, Zhu Q (2019). STAT3-induced upregulation of lncRNA ABHD11-AS1 promotes tumour progression in papillary thyroid carcinoma by regulating miR-1301-3p/STAT3 axis and PI3K/AKT signalling pathway. Cell Prolif.

[34] Gao Y, Zhang Z, Li K, Gong L, Yang Q, Huang X, Hong C, Ding M, Yang H (2017). Linc-DYNC2H1-4 promotes EMT and CSC phenotypes by acting as a sponge of miR-145 in pancreatic cancer cells. Cell Death Dis.

[35] Li T, Qin Y, Zhen Z, Shen H, Cong T, Schiferle E, Xiao S (2019). Long non-coding RNA HOTAIR/microRNA-206 sponge regulates STC2 and further influences cell biological functions in head and neck squamous cell carcinoma. Cell Prolif.

[36] Gu W, Yuan Y, Wang L, Yang H, Li S, Tang Z, Li Q (2019). Long non-coding RNA TUG1 promotes airway remodelling by suppressing the miR-145-5p/DUSP6 axis in cigarette smoke-induced COPD. J Cell Mol Med.

[37] Shen S-N, Li K, Liu Y, Yang C-L, He C-Y, Wang H-R (2019). Down-regulation of long noncoding RNA PVT1 inhibits esophageal carcinoma cell migration and invasion and promotes cell apoptosis via microRNA-145-mediated inhibition of FSCN1. Mol Oncol.

[38] Kong X, Qi J, Yan Y, Chen L, Zhao Y, Fang Z, Fan J, Liu M, Liu Y (2019). Comprehensive analysis of differentially expressed profiles of lncRNAs, mRNAs, and miRNAs in laryngeal squamous cell carcinoma in order to construct a ceRNA network and identify potential biomarkers. J Cell Biochem.

[39] Liu Y, Ye F (2019). Construction and integrated analysis of crosstalking ceRNAs networks in laryngeal squamous cell carcinoma. PeerJ.

